# Learning programme for on-the-job reflection on guideline use in district nursing practice: a protocol for an action research study

**DOI:** 10.1136/bmjopen-2024-090590

**Published:** 2025-03-18

**Authors:** Inge Wolbers, Arjan van Os, Pieterbas Lalleman, Lisette Schoonhoven, Nienke Bleijenberg

**Affiliations:** 1 Proactive Care for Older People Living at Home, HU University of Applied Sciences Utrecht, Utrecht, Netherlands; 2 Institute for Nursing Studies, HU University of Applied Sciences Utrecht, Utrecht, Netherlands; 3 Fontys University of Applied Sciences, Eindhoven, Netherlands; 4 University Medical Center Utrecht, Julius Center for Health Sciences and Primary Care, Utrecht, Netherlands

**Keywords:** Nurses, Protocols & guidelines, EDUCATION & TRAINING (see Medical Education & Training)

## Abstract

**Abstract:**

**Introduction:**

The rising global demand for district nursing care necessitates effective strategies to support evidence-based decision-making. Despite the extensive development of nursing guidelines, adherence by district nursing teams remains suboptimal, revealing a gap between guideline development and daily practice. The Learning And Reflection for Nurses (LEARN) programme aims to bridge this gap by enhancing guideline use and fostering a learning attitude among district nursing teams. This protocol outlines the programme’s development, components and evaluation approach.

**Methods and analysis:**

An action research approach will be used to develop, adjust and evaluate the LEARN programme. The programme includes two interventions: a team-learning intervention focusing on guideline use and collective learning, and a leadership training intervention for district nurses to enhance their impact and role in adopting new knowledge. The team learning intervention will be sequentially deployed and evaluated in four district nursing teams across three care organisations, each lasting approximately 5 months. The leadership training will be conducted and evaluated in two cohorts, each lasting about 5 months. Participation in the team learning intervention requires at least one district nurse to participate in the leadership training. Each intervention will follow an action spiral structure, with learning outcomes from previous interventions carried over to the next. Data will be collected through observations, written reflections, focus groups and questionnaires via a mobile application. Data analysis will occur in two steps: parallel data collection and analysis during the intervention, followed by a longitudinal qualitative approach to identify learning processes over time and evaluate the intervention’s impact.

**Ethics and dissemination:**

Ethics approval has been obtained from the Ethical Committee Research of the HU University of Applied Sciences Utrecht (reference numbers 165-001-2022 and 157-001-2022). Findings will be disseminated continuously throughout the research via workshops, presentations and summary documents for district nurses and their teams, care organisations, strategic policymakers and the academic community.

**STRENGTHS AND LIMITATIONS OF THIS STUDY**

- Two learning interventions will be designed based on action research (AR) theory, using reflection as the primary strategy to induce expansive learning.
- Although this learning intervention is proposed with AR and learning theories, the programme is not set in stone but can be adapted based on learning processes, team dynamics and new insights, which may lead to improved guideline use in daily practice.
- End-user involvement is crucial for achieving the programme’s goals, necessitating significant time and resources to build authentic and trusting relationships.

## Introduction

 The global demand for care delivery at home is increasing owing to a focus on ageing populations, a focus on ageing at home, complex care needs, shorter hospital stays and cost control.^1 2^ Patients with diverse physical and psychological diseases and comorbidities strive to live at home as long as possible. District nurses (European Qualification Framework (EQF) level 6) play a pivotal role in the community and work in teams together with vocational nurses (EQF level 4), certified nurse assistants (EQF level 3), and, in some teams, health and welfare assistants (EQF level 1 and 2).^3^ These teams, with varied staff levels, skill mix and differences in contract hours,^4^ deliver district nursing care—technical, medical, supportive or rehabilitative interventions or personal care to patients at home.^5^ Guidelines can support the teams in care delivery and enhance consistency to maintain or improve quality.

Guidelines are developed to support evidence-based decision-making in nursing, paramedics and medicine^6 7^ and are considered key tools for translating knowledge into daily practice.^8 9^ Nurses, the largest professional group in healthcare, are central to applying guidelines and working on care quality,^10^ and therefore, an increasing number of nursing guidelines have been developed globally^11^ and nationally to support nurses in routine care provision.^12 13^ In the Netherlands, several monodisciplinary nursing guidelines have recently been developed to address nursing problems that are frequently encountered by older patients who live at home, such as loneliness^14^ and caregiver burden.^15^ However, guideline development alone is insufficient for evidence-based decision-making, as adherence by nursing teams remains suboptimal.^9 16^

Active involvement of end-users has proven to be a necessary step in integrating guidelines into daily practice,^17^ and education is often used as a key strategy to engage nurses and enhance guideline use.^8 9^ Jun *et al*^18^ emphasised the importance of education at the start and throughout implementation. Education facilitates understanding routines, procedures and how systems work in a particular context, potentially leading to a change in nurses’ work practice.^19^ For instance, district nursing teams can learn to work with guidelines and adapt elements to their particular context. Although Jun *et al*^18^ mentioned education as essential, their integrative review overlooked the team-based nature of district nursing, where care decisions are team-oriented but executed by individual team members. Thus, education should target both individuals and teams to be effective. Nonetheless, how team-level education contributes to guideline use remains underexplored.

Facilitation is a technique where an individual makes things easier for others.^20^ It can be seen as the active ingredient that aligns the guideline to the teams involved and their work context. Facilitators balance improvement goals with developing teamwork and building capacity.^20^ They help teams learn and adapt their thinking and practices^9 21^ by using various educational strategies, such as reflection, described as ‘looking for the meaning of an event or history’.^22^ Furthermore, nursing leadership is another essential aspect of facilitation and may be important to support guideline use.^23^ Leadership can be seen as a relational practice in which nurses engage with others to set directions, show agency and get things moving.^24^

So far, little is known about how an educational programme can support guideline use in district nursing. We, therefore, designed the LEARN programme: Learning And Reflection for Nurses to enhance district nursing teams working with guidelines and support a learning attitude. This protocol aims to describe the programme’s development and components, as well as our approach to evaluation.

## Methodology

Action research (AR) will be used to develop the learning programme. Before describing the AR design, some underpinning learning theories will be considered.

### Learning theories

Engeströms’ activity theory provides a framework for understanding collective learning and innovation within teams.^19^ In this framework, learning is perceived as ‘expansive learning’, a collective, multivoiced process where an activity system, such as a district nursing team, resolves internal contradictions by creating knowledge and activities.^15^ For instance, district nursing teams discuss and negotiate their care practices for lonely patients, determining which guideline elements are suitable and how to adapt them for daily use. The concept of expansive learning is rooted in Vygotsky’s Zone of Proximal Development,^25^ which emphasises what learners can achieve with the support of others compared with what they can achieve independently. Expansive learning focuses on a community of learners rather than individual learners. According to activity theory, expansive learning results from social-human interactions within specific material, cultural and historical contexts of work practices and drives meaningful change.^26^ Thus, viewing learning as ‘expansive learning’ aligns with a programme that aims to support district nursing teams in how to work with guidelines.

Reflectivity is crucial in AR, and reflection will be emphasised as the primary learning strategy in both learning interventions to catalyse the collaborative exchange of experiences. Reflexivity involves a self-including mental activity in which an individual monitors and reflects on social interactions involving oneself and others and stems from the person’s ongoing internal dialogue.^27 28^ Meta-reflexivity, a specific mode of reflexivity, encompasses professional and private dialogues mingled with others within a social context.^28^ The dialogue, mediated by interpersonal trust and other positive emotions among team members,^29^ is valuable in team and leadership interventions. Furthermore, reflexivity is a powerful learning strategy and a social mechanism. Through repeated human interaction and inner dialogues, individuals can clarify, analyse and refine their understanding of complex difficulties^30^ district nursing teams face, such as loneliness among older people or overburdened caregivers. Moreover, collective reflection as a learning strategy allows sharing and discussing experiences to induce and revive expansive learning cycles.^30 31^

### Design

This study will be designed as a learning intervention to support district nursing teams in developing a learning attitude and contribute to meaningful change in guideline use in daily practice. We will employ an AR approach to develop, adjust and evaluate the LEARN programme. AR is described as social research conducted by a team comprising a professional action researcher and members of an organisation seeking improvement. AR promotes broad participation in the research process and supports actions leading to a more just, sustainable or satisfying situation for stakeholders.^32^ The AR approach is suitable for this study for several reasons. First, by engaging in AR, district nursing team members will be supported in developing a learning attitude and contributing to meaningful change. This bottom-up procedure promotes active collaboration, learning and practical organisational problem-solving.^33^ It comprises a learning process for all involved, making this research an educational intervention.^34^ Additionally, AR allows us to observe and understand how teams learn to work with guidelines in practice.^34^ Finally, the action spirals, with their characteristic phases of (pre-)orientation, planning, action and observation and evaluation and reflection,^34^ will enable us to navigate the learning intervention, making it context-specific.^17^

During pre-orientation, access to a district nursing team in a care organisation will be arranged, the research focus will be determined, information will be shared and agreements with managers and the district nurse will be made. The orientation phase will emphasise relationship building and obtaining approval while examining current and desired work routines around a predetermined care theme. The planning phase will involve defining goals and possible actions. During the acting and observing phase, the team will execute formulated actions. The evaluation and reflection phase will systematically review the process,^34^ followed by possible new actions.

### Setting and participants

This study will be conducted in three district nursing care organisations in the urban environment of Utrecht (360 000 inhabitants), the Netherlands. These organisations are part of a formal regional network (Academische Werkplaats in de wijk) connecting care organisations with the University of Applied Sciences Utrecht and the University Medical Center Utrecht for research and innovation purposes.^35^ Our research focuses on district nursing care teams, particularly district nurses. The teams will be selected with the help of managers from the care organisations, who will also invite district nurses in the designated teams to attend the leadership training. The composition and size of the participating teams depend on the district’s size, population density and demographic characteristics. Another crucial factor that shapes the team composition is the shortage of healthcare professionals,^36^ compelling teams to rely increasingly on temporary nurses, including bank nurses, float nurses, agency nurses and nurse students.

### Patient and public involvement

None.

## Research plan and governance

Two learning interventions will be developed for this study. The main intervention targets district nursing teams, focuses on working with guidelines and supports a collective learning attitude. The second learning intervention supports the first and specifically targets district nurses (EQF 6) from various care organisations and will consist of leadership training to equip them to increase their impact and role in adopting new knowledge. Elements of this programme are derived from a previous nationwide leadership programme for district nurses in the Netherlands.^37^ A mentoring trajectory^38^ and peer-to-peer shadowing^39^ will be added alongside the leadership training.

**Figure 1:**
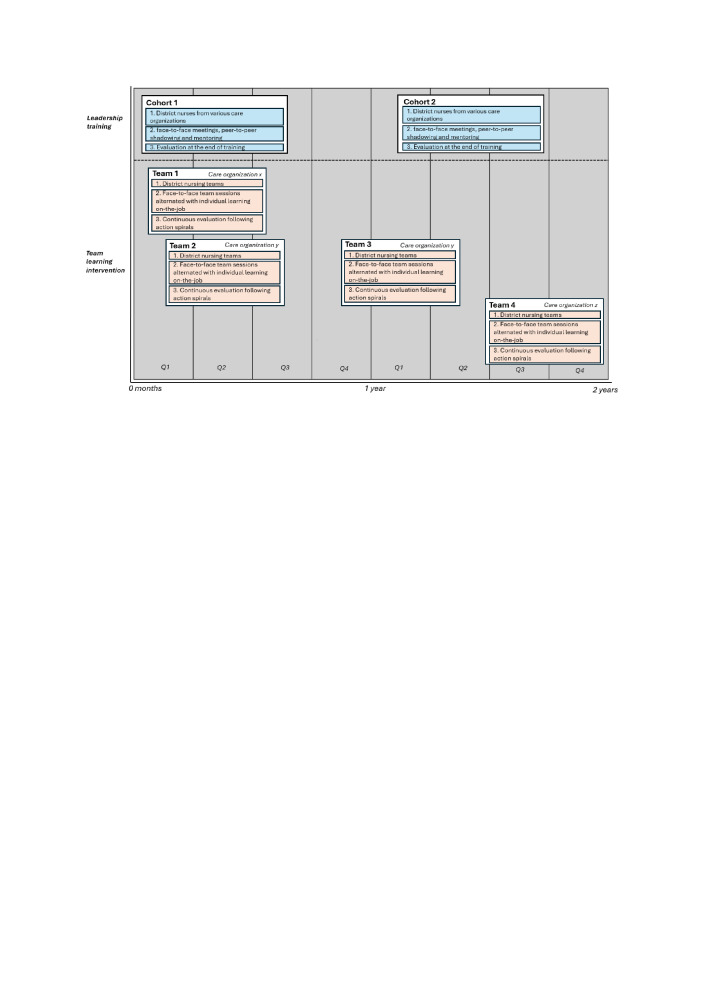
Study overview.

The study will span 45 months, from March 2021 to January 2025. As shown in figure 1, data collection will take 2 years. The team learning intervention will be sequentially deployed and evaluated in four district nursing teams across three care organisations, each lasting approximately 5 months. The leadership training will be conducted and evaluated in two cohorts, each lasting about 5 months. For a team to participate in the learning intervention, at least one district nurse must have participated in the leadership training. Each intervention will follow the structure of an action spiral, with the learning outcomes from the previous intervention carried over to the next.

In the preorientation phase of the team learning intervention, the researchers will collaborate with a local manager in every care organisation to adapt the programme to their context, discuss the research’s practical relevance and address potential issues. During the intervention, following the orientation and subsequent phases of the action spiral, we will work closely with the district nurse participating in the leadership training. The goal is to build relationships, discuss practical matters and use the district nurse’s expertise to plan and develop preparatory materials for the team sessions.

The research team, comprising former nurses who are now professors in proactive eldercare, leadership and nursing science, a PhD candidate and a lecturer in nursing focusing on care ethics, will design the preliminary LEARN programme. During the team learning programme and the leadership training, lecturer AvO and researcher IW will act as facilitators. Concurrently, data collection and analysis will be conducted by the research team and the district nurse. Findings will inform the redesign, the application and the evaluation of the learning interventions.

### Description of the learning interventions

Considering the learning objectives, a team learning programme for district nursing teams and leadership training for district nurses were designed. First, the team learning programme and its content will be described. Second, the leadership training will be outlined, including the individual mentoring trajectory and peer-to-peer shadowing. The initial programmes were designed in spring 2021.

#### The team learning programme

**Figure 2:**
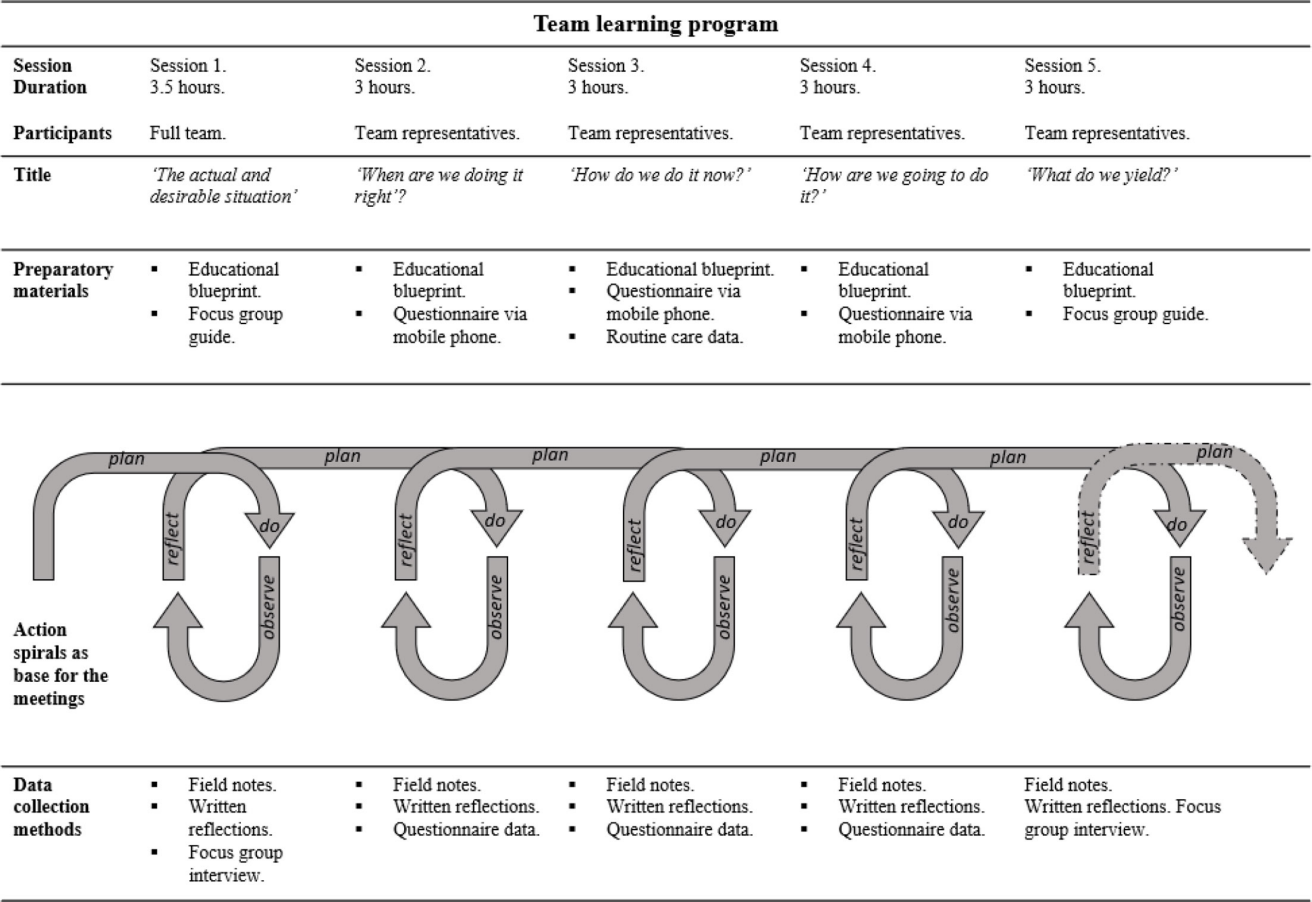
Team learning invention overview combined with data collection methods.

The objective of the team learning programme for district nursing teams is to learn how to work with the guideline ‘loneliness' or 'caregiver burden.' The programme will include five sessions totalling thirteen hours over approximately 5 months. Sessions will be held within the care organisation during working hours to lower participation barriers. The target group includes district nurses, vocational nurses, certified nursing assistants and helping aids. The format combines face-to-face sessions and learning on the job, encouraged by a weekly questionnaire via a mobile phone application. Figure 2 illustrates the programme’s design. Ideally, the entire district nursing team should attend all sessions, but owing to cost constraints and nursing shortages, only the first session will include the whole team. At the end of the first session, a core team of four to six motivated volunteers will be formed to participate in subsequent sessions. This core team will represent the whole team and actively inform other members.

#### Content of the team learning programme

Before the sessions begin, an email detailing the programme’s content and relevant nursing guidelines, along with a video about the guidelines, will be sent to all district nursing team members. This provides an initial overview of the learning programme and the selected guideline. Each session (except the first) will start with a summary of the previous session and end with a preview of the next session, inviting team members to share their input for the next meeting.

The **first** session will address the gap between the actual and desired situations around the guidelines’ care theme. In AR, it is common to investigate the actual situation first, then the desired situation and then explore the gap between them.^34^ The actual situation will be examined through a focus group discussion, asking questions such as whether nurses recognise the guidelines’ care theme in daily work practice, the nursing interventions they typically undertake and the factors that help or hinder them. This will allow facilitators to analyse the team’s perception of the actual situation. Next, the desired situation will be clarified by examining the team members’ underlying values related to the guideline’s subject. The desired situation will be defined by values and reformulated as the team’s ‘collective ambition’.

In the **second** session, the core team will determine the new work standard for the chosen care theme. They will explore the guidelines and described actions, relating them to their actual situation and collective ambition. With this in mind, the team will give practical meaning to the collective ambition and establish a new standard. They will then consider practical steps for the team and the organisation to achieve this standard. The facilitators will support them in examining the nursing guidelines to shape and prioritise these steps.

The **third** session will focus on the steps to reach the new standard. This will include relating parts of their daily work to the guideline, such as documenting in the electronic patient record and learning from it. The marshmallow challenge^40^ will be used to highlight collaboration. Group reflection will provide new insights, leading to additional steps that should be taken to achieve the new standard. If necessary, the team can adjust the collectively formulated standard.

The **fourth** session will focus on future steps and practicalities to help or hinder reaching the team’s new standard. A long-term action plan for the entire district nursing team will be initiated. Using educational methodologies, such as group discussion and mind mapping, the team will explore how the new standard can fit into existing work routines and what steps they can take to align all team members with the new standard.

The **final** session will review the team’s learning achievements. Guided by a focus group using sticky notes and sheets on the wall, the core team will highlight successes and changes in their work regarding the guidelines’ care theme. They will discuss how the guideline’s content is integrated into existing work structures and routines.

##### The leadership training

The leadership training aims to equip district nurses to increase their impact and role in adopting new knowledge. It is hypothesised that the leadership training will work as a catalyst for change, better equipping district nurses to support teams in changing the daily practices around the guidelines ‘loneliness among older people’ or ‘caregivers burden’. Since LEARN is temporary, we aim to sustain the movement for change and equip district nurses to develop leadership practices within their teams or organisations.

**Figure 3:**
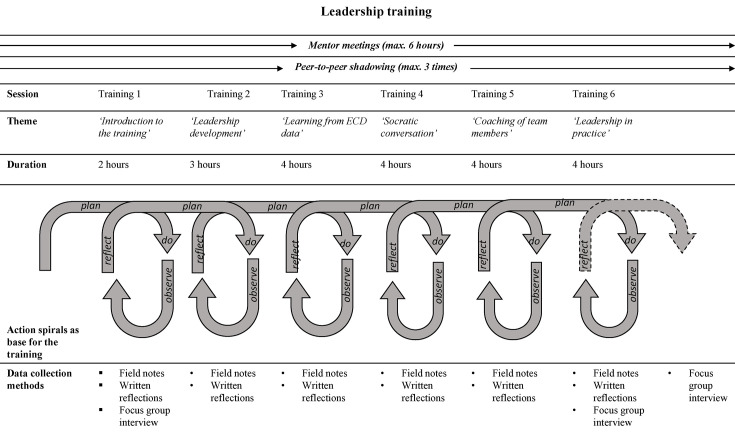
Leadership training overview combined with data collection methods.

The leadership training is a 6-month interorganisational expansive learning programme for district nurses. It includes six face-to-face meetings totalling 20 hours, held outside the organisations to allow nurses to step back from daily practice and create space for learning and reflection. Each meeting will feature an expert exploring a theme using varied educational methodologies and initiating discussions. District nurses will participate in expert meetings, related assignments and group reflections. Figure 3 illustrates the training’s design and content.

The training will begin with the district nurses’ experiences in daily work practice. Meeting content will cover leadership development, learning from patient data, learning from questioning and coaching team members. Although content areas are predetermined, the educational methodology will be tailored to the group’s learning needs. The interorganisational nature offers opportunities for mutual enrichment.

##### Individual mentoring trajectory

Alongside the leadership training, an individual mentoring trajectory will be initiated, inspired by Hafsteinsdóttir *et al*.^38^ The goal is to support the ongoing leadership development of district nurses. The individual mentoring trajectory will be guided by the district nurses’ actual learning needs, with each nurse meeting their mentor six times (6 hours). The project team will match each nurse with a mentor who is an expert in nursing and works as an educator or researcher in district nursing care, long-term care or has a coaching affinity. Mentors will be informed about the leadership training but will not receive additional education to shape the mentoring trajectory.

##### Peer-to-peer shadowing

The leadership training includes peer-to-peer shadowing to encourage mutual enrichment and learning. This method focuses on variations in daily practice and clinical leadership, reflection on roles, problem-solving strategies and triggering learning processes that support leadership development. Lalleman *et al*^39^ found that peer-to-peer shadowing facilitates collective reflection-in-action and enhances an ‘investigate stance’ for both the observer and the observed. In the leadership training, peer-to-peer shadowing will occur at three different times. The first encounter will be in meeting two, where participants receive information and instruction. The first shadowing session will last 1 hour, with participants shadowing a peer from a different organisation during a moment with multiple contacts. The second session, with the same peer, will last half a workday. The third session, with a different peer from another organisation, will also last half a workday.

## Evaluation

The evaluation will involve all qualitative data collected from March 2021 to January 2025. Qualitative methods will provide a better understanding of the contexts, how the learning programme unfolds and identify relevant hindrances or helping factors for working with guidelines. As the learning interventions occur in a short time frame, data will be quickly analysed and used as input for the next session. For the final analysis, a longitudinal qualitative approach will be adopted.

### Data collection

In the team learning intervention and the leadership training, data will be gathered through various methods. During all phases of the action spirals, data will be collected through shadowing^41^ to understand how district nursing teams learn to work with the guidelines ‘loneliness among older people’ or ‘caregiver burden’. Observations during sessions will focus on dialogue, verbalised perceptions and participation. Written notes (ie, keywords, situations, quotes, Head2 short sentences)^42^ will be structured into fieldnote reports and treated as data. After each session, the researcher and the nurse lecturer (IW, AvO) will write reflections to understand the observations, serving as a diary. Focus groups will be used at the start and end of both interventions to explore experiences with loneliness, caregiver burden and guideline use and evaluate the impact of the learning interventions. Focus groups enable in-depth team discussion and effectively explore values, beliefs and systems.^43^ In the team learning programme, a brief, tailored questionnaire will be distributed via a mobile phone application^44^ following the time-based sampling method^45^ during the weeks between sessions. This diary method will provide insight into team members’ learning moments on the job and the application of the guideline. At the beginning of the second, third and fourth team sessions, some answers will be highlighted to spark conversations about current work situations as cases during the sessions.

### Data analysis

Data analysis will occur in two steps. First, data will be collected and analysed in parallel, evolving with the learning intervention. Collaborative analysis with the facilitators (IW, AvO), the district nurse and the research team (PL, LS, NB) will take place during meetings, providing the foundation for reflection on patterns, underlying causes and themes.^46^ This reflection, part of the action spiral,^30^ will inform the preparatory materials for subsequent sessions. Second, a longitudinal qualitative approach^47^ will be used to identify the unfolding of learning processes over time in a district nursing team and evaluate the intervention’s impact. We will reconstruct the timeline and group the related observational reports, reflections and transcripts. The analysis and sense-making will follow the six steps described by Braun and Clarke.^48^

### Ethics and safety

The study has been approved by the Ethical Committee Research of the University of Applied Sciences Utrecht (reference numbers 165-001-2022 and 157-001-2022). All participants will be asked to provide their written consent before participating in either the team learning programme or the leadership training. All participants will be informed that they can withdraw from the study at any time.

### Dissemination plan

Dissemination will be a continuous process throughout the research. Workshops, presentations and summary documents will be developed for different audiences. A symposium will be organised for the participating organisations and their staff to summarise our research activity and output, providing guidance on incorporating expansive learning in daily practice. User-friendly versions of our findings will be developed for the Dutch nursing audience, care organisations and policy makers. For the academic community, research articles in peer-reviewed journals will be written, and conference presentations will be held. Preliminary findings will also be regularly presented within the research centres at the University of Applied Sciences Utrecht and University Medical Center Utrecht.

## Discussion

This article outlines the LEARN programme, a research study and educational intervention for district nurses. It supports district nursing teams in learning how to use the guideline ‘loneliness among older people’, or ‘caregiver burden’ in daily work practice, and it comprises a leadership programme to enhance district nurses’ impact in adopting new knowledge. Nevertheless, this design has potential challenges.

First, the protocol’s strong emphasis on theoretical concepts and design for both the team learning programme and the leadership training may be seen as a set programme that cannot be altered. This may contradict AR principles.^32^ However, the design outlined here is preliminary, and alternating phases of data collection and analysis will inform the next steps, making it responsive to context.^17^ Moreover, the design enables researchers to go beyond improving practice and develop theories on strategies that enhance district nursing teams’ guideline use in daily practice.^49^

Second, the LEARN programme relies on end-user involvement, including district nurses and district nursing teams, and is a prerequisite for achieving the programme’s goals. Therefore, time and resources are required to build authentic and trusting relationships between the research team and the end users.^50^ The pitfall will be prioritising speed over engagement and collaboration.^17^

Although this study protocol focuses on improving guideline use in district nursing practice, some elements of the LEARN programme may be transferred to allied health practices. A systematic review by Goorts *et al*^51^ showed that an implementation strategy for allied healthcare professionals to use guidelines should be adaptive to the context, include educational meetings and support clinicians. Therefore, the team learning programme and the leadership training may also be converted to other health professionals.
