# Supplementary material for: Learning programme for on-the-job reflection on guideline use in district nursing practice: a protocol for an action research study
Source: BMJ Open. 2025 Mar 18;15(3):e090590. doi: 10.1136/bmjopen-2024-090590 (PMC11927463; doi:10.1136/bmjopen-2024-090590)
Supplement: Supplementary file 1 [file bmjopen-2024-090590.reviewer_comments.pdf]

PEER REVIEW HISTORY

BMJ Open publishes all reviews undertaken for accepted manuscripts. Reviewers are asked to complete a checklist review form and are provided with free text boxes to elaborate on their assessment. These free text comments are reproduced below.

ARTICLE DETAILS

Title (Provisional)

PROTOCOL FOR AN ACTION RESEARCH STUDY: A LEARNING PROGRAMME FOR ON-THE-JOB REFLECTION ON GUIDELINE USE IN DISTRICT NURSING PRACTICE.

Authors

Wolbers, Inge; van Os, Arjan; Lalleman, Pieterbas; Schoonhoven, Lisette; Bleijenberg, Nienke

---

VERSION 1 - REVIEW

**Reviewer**	**1**
**Name**	**Morris, Meg**
**Affiliation**	**La Trobe Univ, Room 423 HS3**
**Date**	**27-Oct-2024**
**COI**	**None**

This unique and innovative project is on a clinically relevant topic - the need to find ways to optimize the use of clinical guidelines in nurses and other health professionals. Major revisions are required:

1. The abstract and the manuscript need to more clearly state the research question and describe the theoretical models used in the qualitative analysis.

2. The main manuscript is too lengthy and the introduction and discussion both need to be reduced by around one-third and at the same time refer more extensively to recent publications (eg last 5 years) from other research groups around the world. The introduction also needs to cite some specific recent clinical guidelines to illustrate why health professionals should be using them.

3. The methods needs to more clearly describe the design.

4. The method needs to more clearly describe and justify the exact qualitative methods to be used. As part of this the Spiral Process needs to be defined and justified more clearly.

5. The analysis of qualitative results needs to be detailed more comprehensively and i currently unclear.

6. The figures are hard to follow - please remove the blue coloring and layering and make the text content more clear.

7. The discussion does not adequately refer to recent research from other groups across the world.

8. The discussion needs to describe more comprehensively the implications and impact for midwifery, allied health and medicine.

9. The discussion needs to make reference to some specific recent clinical guidelines

10. The limitations section needs more detail.

11. The conclusion is far too long and repetitious. Please replace it with 2-3 new sentences.

12. The reference list contains a lot of unnecessary old manuscript yet misses important recent clinical guidelines, such as the world falls guidelines.

**Reviewer**	**2**
**Name**	**Nikpeyma, Nasrin**
**Affiliation**	**TUMS**
**Date**	**24-Nov-2024**
**COI**	**None**

The article is very long

It is better to write the type of action research and its stages separately and specify exactly what the researcher does at each stage

Specify the data analysis method

The duration of action research is long

VERSION 1 - AUTHOR RESPONSE

REVIEWER #1 COMMENTS:

0. *This unique and innovative project is on a clinically relevant topic - the need to find ways to optimise the use of clinical guidelines in nurses and other health professionals. Major revisions are required.*
   RESPONSE:
   We thank the reviewer for the positive comment and the thorough feedback. We responded to the comments as adequately as possible.

1. *The abstract and the manuscript need to more clearly state the research question and describe the theoretical models used in the qualitative analysis.*
   RESPONSE:

We transformed the abstract into the prescribed format. We clarified the research question and the theoretical models. Please see the response to Editor comment number 2.

2. *The main manuscript is too lengthy, and the introduction and discussion both need to be reduced by around one-third and, at the same time, refer more extensively to recent publications (e.g. last 5 years) from other research groups around the world. The introduction also needs to cite some specific recent clinical guidelines to illustrate why health professionals should be using them.*

   RESPONSE:

   We agree that the manuscript is lengthy. We reduced both the introduction and the discussion. Initially, the introduction contained 952 words and we reduced it to 570 words.

   <u>Page 4, line 87 – Page 6, line 127.</u>

"The global demand for care delivery at home is increasing due to a focus on ageing populations, a focus on ageing at home, complex care needs, shorter hospital stays, and cost control.(1, 2) Patients with diverse physical and psychological diseases and comorbidities strive to live at home as long as possible. District nurses (European Qualification Framework [EQF] level 6) play a pivotal role in the community and work in teams together with vocational nurses (EQF level 4), certified nurse assistants (EQF level 3), and, in some teams, health and welfare assistants (EQF level 1 and 2).(3) These teams, with varied staff levels, skill mix, and differences in contract hours (4), deliver district nursing care – technical, medical, supportive, or rehabilitative interventions or personal care to patients at home.(5) Guidelines can support the teams in care delivery and enhance consistency to maintain or improve quality.

Guidelines are developed to support evidence-based decision-making in nursing, paramedics, and medicine.(6, 7) and are considered key tools for translating knowledge into daily practice.(8, 9) Nurses, the largest professional group in healthcare, are central to applying guidelines and working on care quality (10), and therefore, an increasing number of nursing guidelines have been developed globally (11) and nationally to support nurses in routine care provision.(12, 13) In the Netherlands, several monodisciplinary nursing guidelines have recently been developed to address nursing problems that are frequently encountered by older patients who live at home, such as loneliness.(14) and caregiver burden.(15) However, guideline development alone is insufficient for evidence-based decision-making, as adherence by nursing teams remains suboptimal.(16, 9)

Active involvement of end-users has proven to be a necessary step in integrating guidelines into daily practice.(17), and education is often used as a key strategy to engage nurses and enhance guideline use.(8, 9) Jun et al. (18) emphasized the importance of education at the start and throughout implementation. Education facilitates understanding routines, procedures, and how systems work in a particular context, potentially leading to a change in nurses' work practice.(19). For instance, district nursing teams can learn to work with guidelines and adapt elements to their particular context. Although Jun et al. (18) mentioned education as essential, their integrative review overlooked the team-based nature of district nursing, where care decisions are team-oriented but executed by individual team members. Thus, education should target both individuals and teams to be effective. Nonetheless, how team-level education contributes to guideline use remains underexplored.

Facilitation is a technique where an individual makes things easier for others.(20) It can be seen as the active ingredient that aligns the guideline to the teams involved and their work context. Facilitators balance improvement goals with developing teamwork and building capacity.(20) They help teams learn and adapt their thinking and practices (9, 21) by using various educational strategies, such as reflection, described as "looking for the meaning of an event or history".(22, 23) Furthermore, nursing leadership is another essential aspect of facilitation and may be important to support guideline use.(24) Leadership can be seen as a

relational practice in which nurses engage with others to set directions, show agency, and get things moving.(25)

So far, little is known about how an educational programme can support guideline use in district nursing. We, therefore, designed the LEARN programme: Learning And Reflection for Nurses to enhance district nursing teams working with guidelines and support a learning attitude. This protocol aims to describe the programme's development and components, as well as our approach to evaluation."

We specifically cited other recent clinical guidelines in the introduction.

Page 19, lines 464 – 471.

- "Montero-Odasso M, Van Der Velde N, Martin FC, Petrovic M, Tan MP, Ryg J, et al. World guidelines for falls prevention and management for older adults: a global initiative. *Age Ageing.* 2022;51(9):afac205."
- "Dutch Nursing Association [V&VN]. *Guideline for addressing loneliness in older adults Loneliness [Richtlijn Eenzaamheid onder ouderen].* Utrecht: V&VN. Available from https://kennisplatform.venvn.nl/onderwerp/eenzaamheid/"
- "Dutch Nursing Association [V&VN]. *Guideline caregiver burden [*Richtlijn Mantelzorg*]*. Utrecht: V&VN. Available from: https://kennisplatform.venvn.nl/onderwerp/mantelzorg/"

We reduced the discussion to 300 words.

Page 17, lines 410 – 432.

"This article outlines the LEARN programme, a research and educational intervention for district nurses. It describes the development of the LEARN programme for district nurses and their teams, which is a research programme and an educational intervention simultaneously. It supports district nursing teams in learning how to use the guideline 'loneliness,' or caregivers burden' in daily work practice, and it comprises a leadership programme to enhance district nurses' impact in adopting new knowledge. Nevertheless, this design has potential challenges.

First, this protocol, with a strong emphasis on the theoretical concepts and design for both the team learning programme and the leadership training may be interpreted as a set programme that cannot be altered. This may contradict action research principles.(35) However, the design outlined here is preliminary, and the alternating phases of data collection and analysis will inform the next steps, making it responsive to context.(17) Moreover, the design enables researchers to go beyond improving practice and develop theories on strategies that enhance district nursing teams' guideline use in daily practice.(52)

Second, the LEARN programme relies on end-user involvement, including district nurses and district nursing teams, and is a prerequisite for achieving the programme's goals. Therefore, time and resources are required to build authentic and trusting relationships between the research team and the end users.(53) The pitfall will be prioritizing speed over engagement and collaboration.(17)

Although this study protocol focuses on improving guideline use in district nursing practice, some elements of the LEARN programme may be transferred to allied health practices. A systematic review by Goorts, Dizon, and Milanese (54) showed that an implementation strategy for allied healthcare professionals to use guidelines should be adaptive to the context, comprise educational meetings, and support clinicians. Therefore, the team learning programme and the leadership training may also be converted to other health professionals."

3. *The methods needs to more clearly describe the design.*

RESPONSE:

We now provide a clearer description of the design by highlighting the advantages of conducting Action Research and offering a more detailed explanation of the application of its cyclic phases.

Page 7, lines 173 - 199.

"This study will be designed as a learning intervention to support district nursing teams in developing a learning attitude and contribute to meaningful change in guideline use in daily work practice. We will choose an Action Research (AR) approach to develop, adjust, and evaluate the LEARN programme. *"AR can be described as social research carried out by a team that encompasses a professional action researcher and members of an organisation seeking to improve the situation. AR promotes broad participation in the research process and supports action leading to a more just, sustainable, or satisfying situation for the stakeholders"* .(35) The AR approach is suitable for this study for several reasons. First, by 'doing' AR, members of district nursing teams are supported to develop a learning attitude and contribute to meaningful change. This bottom-up procedure promotes active collaboration, learning, and practical organisational problem-solving.(36) It comprises a learning process for everybody involved, and it makes this research, thus, an educational intervention.(37) Additionally, AR allows us to observe and understand the process of how teams learn to work with a guideline while working.(37) Finally, the action spirals with the characteristic consecutive phases of (pre-)orientation, planning, action and observation, and evaluation and reflection (37) will enable us to navigate the learning intervention, making it context-specific.(17)

The learning intervention follows a cyclic process involving (pre-)orientation, followed by planning, acting, observing, evaluating, and reflecting.(37) During preorientation, access to a district nursing team in a care organisation will be arranged, the research focus will be determined, information about the research will be shared, and agreements with managers and the district nurse will be made. The orientation phase will emphasise relationship building with the district nursing team and obtaining approval. Also, the current and desired work routine around a pre-determined care theme will be examined. The planning phase will involve defining goals and possible actions. In the acting and observing phase, the team will execute formulated actions. The process will be systematically reviewed in the evaluation and reflection phase (37), and possible new actions will be planned."

4. *The method needs to more clearly describe and justify the exact qualitative methods to be used. As part of this the Spiral Process needs to be defined and justified more clearly.*

RESPONSE:

We described and justified the qualitative research methods in the evaluation section.

Page 15, lines 363 – 366.

"In the team learning intervention and the leadership training, data will be gathered through diverse methods. During all the phases of the action spirals, data will be collected through shadowing. (44) It provides an understanding of how district nursing teams learn to work with the guideline 'loneliness' or 'caregiver burden."

Page 15, lines 375 – 382.

"We will use focus groups at the start and end of both interventions to explore experiences with loneliness, caregiver burden, and guideline use, and evaluate the impact of the learning interventions. Focus groups enable in-depth team discussion and effectively explore values, beliefs, and systems.(46) In the team learning programme, we will additionally distribute a brief, tailored questionnaire through a mobile phone application (47) following the time-based sampling method (48) during the weeks between the sessions. This diary method will provide insight into team members' learning moments on the job and the appliance of the guideline."

We agree that the cyclic spiral process needs further definition and justification. Therefore, we described the action spirals in more detail in the design section mentioned in the previous response (comment number 3, reviewer 1).

5. *The analysis of qualitative results needs to be detailed more comprehensively and is currently unclear.*

   RESPONSE:

   We described the data analysis in more detail.

   Page 16, lines 390 – 399.

"Data analysis occurs in two steps. First, data will be collected and analyzed in parallel, gradually evolving along the learning intervention. Collaborative analysis with the facilitators [IW, AVO], the district nurse, and the research team [PL, LS, NB] will take place during various meetings and provide the foundation for reflection on patterns, underlying causes, and themes.(49) As the reflection is part of the action spiral (33), this informs the preparatory materials for the next team learning session and leadership training. Second, we will use a longitudinal qualitative approach (50) to identify the unfolding of learning processes over time in a district nursing team and evaluate the impact of this learning intervention. We will reconstruct the timeline and group the related observational reports, reflections, and transcripts. The analysis and sensemaking will follow the six steps described by Braun & Clarke.(51)"

6. *The figures are hard to follow - please remove the blue coloring and layering and make the text content more clear.*

   RESPONSE:

   Regarding Figure 1, we removed the dark blue colouring and the layering. Instead, we used higher contrast in the figure. To avoid the layering, we added a timeline to be more clear on the intended planning. Moreover, we elaborated on the text content.

   Regarding Figures 2 and 3, we removed the blue colouring.

7. *The discussion does not adequately refer to recent research from other groups across the world.*

   RESPONSE:

   We added references to the discussion that refer to recent research.

8. *The discussion needs to describe more comprehensively the implications and impact for midwifery, allied health and medicine.*

   RESPONSE:

   We described the possible implications and impact for allied health professionals.

   Page 17, lines 427 – 432.

"Although this study protocol focuses on improving guideline use in district nursing practice, some elements of the LEARN programme may be transferred to allied health practices. A systematic review by Goorts, Dizon, and Milanese (54) showed that an implementation strategy for allied healthcare professionals to use guidelines should be adaptive to the context, comprise educational meetings, and support clinicians. Therefore, the team learning programme and the leadership training may also be converted to other health professionals."

9. *The discussion needs to make reference to some specific recent clinical guidelines.*

RESPONSE:

We acknowledge the importance of clinical guidelines. However, this manuscript focuses on how district nurses and their teams learn how to work with guidelines in daily practice. Therefore, we do not make reference to some specific clinical guidelines in the discussion section but focus on the learning interventions. Nevertheless, we did refer to some clinical guidelines in the introduction.

10. *The limitations section needs more detail.*

    RESPONSE:

    We have shortened the discussion and prioritized less theoretical and more specific limitations. <u>Page 17, lines 416 – 426.</u>

"First, this protocol, with a strong emphasis on the theoretical concepts and design for both the team learning programme and the leadership training may be interpreted as a set programme that cannot be altered. This may contradict action research principles.(35) However, the design outlined here is preliminary, and the alternating phases of data collection and analysis will inform the next steps, making it responsive to context.(17) Moreover, the design enables researchers to go beyond improving practice and develop theories on strategies that enhance district nursing teams' guideline use in daily practice.(52)

Second, the LEARN programme relies on end-user involvement, including district nurses and district nursing teams, and is a prerequisite for achieving the programme's goals. Therefore, time and resources are required to build authentic and trusting relationships between the research team and the end users.(53) The pitfall will be prioritizing speed over engagement and collaboration.(17)"

11. *The conclusion is far too long and repetitious. Please replace it with 2-3 new sentences.*

    RESPONSE:

    The editor advised us to remove the conclusion as this is not a part of the journal formatting requirements for protocol articles. Therefore, we removed the conclusion.

12. *The reference list contains a lot of unnecessary old manuscript yet misses important recent clinical guidelines, such as the world falls guidelines.*

    RESPONSE:

We removed the unnecessary old manuscripts and updated the reference list with recent manuscripts. We mentioned some clinical guidelines in the introduction, including the World Falls Guidelines, and added them to the reference list. Please see the changes in the reference list.

REVIEWER #2 COMMENTS:

1. *The article is very long.*

   RESPONSE:

   We shortened the article by reducing both the introduction and the discussion.

2. *It is better to write the type of action research and its stages separately and specify exactly what the researcher does at each stage.*

   RESPONSE:

We agree. In the design section, we described the stages separately and specified on the researchers' actions.

Page 8, lines 188 – 197.

"The learning intervention follows a cyclic process involving (pre-)orientation, followed by planning, acting, observing, evaluating, and reflecting.(37) During preorientation, access to a district nursing team in a care organisation will be arranged, the research focus will be determined, information about the research will be shared, and agreements with managers and the district nurse will be made. The orientation phase will emphasise relationship building with the district nursing team and obtaining approval. Also, the current and desired work routine around a pre-determined care theme will be examined. The planning phase will involve defining goals and possible actions. In the acting and observing phase, the team will execute formulated actions. The process will be systematically reviewed in the evaluation and reflection phase (37), and possible new actions will be planned."

3. *Specify the data analysis method.*

   RESPONSE:

   We specified the data analysis method.

   Page 15, lines 382 – 391.

   Data analysis occurs in two steps. First, data will be collected and analyzed in parallel, gradually evolving along the learning intervention. Collaborative analysis with the facilitators [IW, AVO], the district nurse, and the research team [PL, LS, NB] will take place during various meetings and provide the foundation for reflection on patterns, underlying causes, and themes.(49) As the reflection is part of the action spiral (33), this informs the preparatory materials for the next team learning session and leadership training. Second, we will use a longitudinal qualitative approach (50) to identify the unfolding of learning processes over time in a district nursing team and evaluate the impact of this learning intervention. We will reconstruct the timeline and group the related observational reports, reflections, and transcripts. The analysis and sensemaking will follow the six steps described by Braun & Clarke.(51)

4. *The duration of action research is long.*

   RESPONSE:

   The entire study will take 45 months. Each cohort of the leadership training will take 6 months. Each team learning intervention will take approximately five months.

   Page 9, lines – 216 – 219.

   "The entire study will be undertaken in 45 months, from March 2021 to January 2025. As illustrated in Figure 1, the total duration of the data collection process will take two years. The team learning intervention will be sequentially deployed and evaluated in four district nursing teams in three different care organisations. The duration of one team learning intervention will be approximately five months."

VERSION 2 - REVIEW

**Reviewer**	**1**
**Name**	**Morris, Meg**
**Affiliation**	**La Trobe Univ, Room 423 HS3**
**Date**	**06-Feb-2025**

COI

Much improved and much more thorough, thank you. This is a very interesting article.

1. There is now overuse of the word "we" throughout - please start many of those sentences with "The" to make the manuscript more readable.

2. Some of the sections are now too long and wordy. Please cut back the non-essential detailing, such as: "Considering the learning objectives, the theoretical frameworks, and our previously gained insight about a leadership development programme for district nurses, we designed a team learning programme for district nursing teams and leadership training for district nurses. We will first describe the team learning programme and zoom in on the content. Second, the leadership training will be described, and the individual mentoring trajectory and peer-to-peer shadowing will be addressed accordingly. The initial programmes were designed in the spring of 2021."

3. The reference list still contains too many old references - please try and remove at least 5 of the outdated ones before 2025.

4. Grammar and expression needs to be checked again please and optimised throughout.

VERSION 2 - AUTHOR RESPONSE

REVIEWER #1 COMMENTS:

1. *Much improved and much more thorough, thank you. This is a very interesting article.*
   RESPONSE: Thank you.

2. *There is now overuse of the word "we" throughout - please start many of those sentences with "The" to make the manuscript more readable.*
   RESPONSE: We minimised the use of 'we' throughout the manuscript. Please see the marked-up version of the manuscript for the textual improvements.

3. *Some of the sections are now too long and wordy. Please cut back the non-essential detailing, such as: "Considering the learning objectives, the theoretical frameworks, and our previously gained insight about a leadership development programme for district nurses, we designed a team learning programme for district nursing teams and leadership training for district nurses. We will first describe the team learning programme and zoom in on the content. Second, the leadership training will be described, and the individual mentoring trajectory and peer-to-peer shadowing will be addressed accordingly. The initial programmes were designed in the spring of 2021."*

RESPONSE: We made additions throughout the manuscript to shorten sections and make it less wordy. Please see the marked-up version manuscript for the textual improvements.

For an example, please see Page 10, lines 231 – 235:

“Considering the learning objectives, a team learning programme for district nursing teams and leadership training for district nurses were designed. First, the team learning programme and its content will be described. Second, the leadership training will be outlined, including the individual mentoring trajectory and peer-to-peer shadowing. The initial programmes were designed in spring 2021.”

4. *The reference list still contains too many old references - please try and remove at least 5 of the outdated ones before 2025.*

   RESPONSE: We removed five of the outdated references.

- ~~Van Eenoo L, Declercq A, Onder G, Finne-Soveri H., Garms-Homolova V, Jonsson PV, et al. Substantial between-country differences in organizing community care for older people in Europe – a review. *Eur J Public Health.* 2016;26(2):213-219.~~
- ~~Heidegger M. *Basic questions of philosophy: selected "problems" of "logic".* Bloomington: Indiana University Press; 1994.~~
- ~~Wald HS. Refining a definition of reflection for the being as well as doing the work of a physician. *Med Teach.* 2015;37(7):1-4.~~
- ~~Engeström Y. Activity theory as a framework for analysing and redesigning work. *Ergonomic.* 2000;43(7):960-974.~~
- ~~Archer MS. *Making our way through the world: human reflexivity and social mobility.* Cambridge: Cambridge University Press; 2007~~

We added two recent references and updated the reference list:

Page 17, lines 413 – 415:

“Veldhuizen JD, Hafsteinsdóttir, TB, Mikkers MC, Bleijenberg N, Schuurmans MJ. Evidence-based interventions and nurse-sensitive outcomes in district nursing care: A systematic review. *Int J Nurs Stud Ad.* 2021;3:100053.”

Page 19, lines 460 – 462:

“Schaepkens SP, Veen M, De La Croix A. Is reflection like soap? A critical narrative umbrella review of approaches to reflection in medication education research. *Adv Health Sci Educ.* 2022;27(2):537-51.”

5. *Grammar and expression needs to be checked again please and optimised throughout.*

RESPONSE: We checked grammar and expression and optimised the manuscript throughout. Please see the marked-up version manuscript for the improvements.
